# Analysis on the alterations of lens proteins by *Vitex negundo* in selenite cataract models

**Published:** 2011-05-06

**Authors:** B.N. Rooban, V. Sasikala, V. Sahasranamam, Annie Abraham

**Affiliations:** 1 Department of Biochemistry, University of Kerala, Kariavattom, Thiruvananthapuram, India; 2 Regional Institute of Ophthalmology, Medical College, Thiruvananthapuram, India

## Abstract

**Purpose:**

Cataract is the leading cause of blindness and is associated with oxidative damage and protein modification in the lens. In the present study, we have employed proteomic and microscopic approaches to investigate the attenuation of selenite cataract by the flavonoids from *Vitex negundo* (FVN).

**Methods:**

To demonstrate this attenuation, Sprague-Dawley rat pups were divided into control (G I), selenite induced (G II), and selenite + FVN treated (G III). Cataract was induced by single subcutaneous injection of sodium selenite (4 mg/Kg bodyweight) on the 10th day and FVN (1 mg/Kg bodyweight) administered intraperitoneally from the 8th to the 15th day.

**Results:**

Our study indicated that chaperone property of α-crystallin and soluble protein levels were reduced in the selenite induced group. Post translational modifications identified by two dimensional-polyacrylamide gel electrophoresis (2D-PAGE) and immunoblot analysis revealed the loss of cytoskeletal proteins in selenite induced group. Damage of lenticular membrane and abnormal fiber structure were observed by electron microscopy.

**Conclusions:**

The results of this study suggest that FVN modulated selenite induced cataractogensis in rat pups by preventing loss of chaperone property, various changes in lens proteins, and lens structure, further strengthening its protective role.

## Introduction

Cataract is the loss of eye lens transparency and one of the most frequent causes of human blindness world wide [1]. It represents a large financial burden on health care systems and there remains a need to develop effective pharmaceuticals for the prevention and treatment [2]. There is no single universally accepted pharmacological agent that has emerged to either inhibit or reverse the progression of cataract and the only treatment is surgery. Also even after surgery, some post operative complications might occur such as posterior capsular opacity, glaucoma, retinal detachment, endophthalmitis, uveitis etc [3,4]. Hence, it is important to look into alternative pharmacological measures for the treatment of this disorder.

Lens comprises three major structural proteins known as crystallins (α, β, and γ), which contribute to its transparency and provide the needed refractive power to focus light on to the retina [5]. Previous studies showed that post translational modifications and accumulation of large amount of insoluble proteins derived from soluble protein due to aggregation is the major mechanism in cataractogenesis [5,6]. Lens cytoskeletal proteins comprise of 2%–4% of the total lens proteins, which include vimentin, tubulin, spectrin, actin etc [7]. The transparency of the crystalline lens has been attributed to the complex, ordered arrangement of its components at both microscopic and molecular levels [8]. Understanding of lens morphology is essential as a basis for characterizing cataract formation and various changes in accommodative ability.

Our experimental model, selenite-overdose cataract is an extremely rapid and convenient model of nuclear cataract in vivo [9]. The mid-stage events producing the cataract include ROS generation, oxidative damage of the critical sulfhydryl groups of membrane Ca^2+^ATPase, calpain mediated proteolysis, precipitation of fragmented lens crystallins, and cytoskeletal proteins [10]. Various medicinal plants and their bioactive components have been reported to render protective effects against selenite cataract, by virtue of its antioxidant effect by our laboratory [11-14] as well as others [15-17]. *V. negundo*, a deciduous shrub belonging to the family of Verbenaceae, is reported to have anti-inflammatory, ophthalmic, analgesic and anti-histaminic properties [18].

This study is an extension of our previously reported work on the attenuation of calpain activation by *V. negundo* in selenite cataract [13]. Proteomics is a valuable tool in elucidating changes in the complex biologic system that involves large network of proteins [19]. In the present study, we aimed to determine the changes in lens proteins in selenite cataract by *V. negundo* administration. The study assumes importance as it is the first report on the effect of flavonoids from *Vitex negundo* (FVN) to protect lens proteins in selenite cataract.

## Methods

### Chemicals and solvents

All the biochemicals and quercetin were purchased from Sigma Chemical Company (St. Louis, MO) and other chemicals and solvents of analytical grade were from SRL Chemicals (New Delhi, India). Protein markers for sodium dodecyl sulphate-polyacrylamide gel electrophoresis (SDS–PAGE) was purchased from GENEI (Bangalore, India).

### Extraction, isolation, and estimation of flavonoids

Fresh leaves of *V. negundo* were collected from Trivandrum, India during early summer. A specimen of the collected material was verified with the herbarium of Tropical Botanical Garden and Research Institute, Thiruvananthapuram, India (Accession number17038; P.S. Jyothish) and authenticated by an expert. The shade dried plant material –leaves (1 Kg) was crushed for extraction. This was taken in a round-bottomed flask, 80% methanol added to cover the material and refluxed in a water bath at 65 °C for 24 h. The supernatant was removed and the extraction was repeated twice. The extract was decanted, filtered, and concentrated to remove the solvent in a rotor evaporator. Extract was cleared off low polarity contaminants such as fats, terpenes, chlorophyll and xanthophyll by repeated extraction with petroleum ether (60– 80 °C), Benzene, and Ethyl acetate, respectively. Ethyl acetate extract contained a bulk of polyphenols (59 g/Kg), which was evaporated in vacuum and the flavonoid content (405 mg/Kg) was determined by using quercetin as reference standard [20]. Flavonoids obtained was dissolved in phosphate-buffered saline (prepared in sterile water) and used for further studies.

### In vivo experiments

Neonatal rat pups of the Sprague-Dawley strain initially weighing 10–12 g on the 8th day of age were used for the study. The pups were housed along with their mother in polypropylene cages in rooms maintained at 25±1 °C. The animals were maintained on a standard laboratory animal diet (Hindustan Lever Ltd., Mumbai, India) and provided water ad libitum throughout the experimental period. Animals were grouped as G I Control (normal laboratory diet), G II (normal laboratory diet + sodium selenite), and G III (normal laboratory diet + sodium selenite + FVN) with eight rats in each group. The neonatal rat pups in experimental groups (II and III) received a single subcutaneous injection of sodium selenite (4 mg/Kg bodyweight) on the 10th day of age. G III received intraperitoneal injection of FVN at the concentration 1.0 mg/Kg bodyweight from the 8th day up to the 15th day of age. Cataract could be visualized from the 16th day of age with the help of an ophthalmoscope and later on with the naked eye. On the 30th day of age, rats were euthanized by sodium pentothal injection, lenses were excised and the experiments conducted. All ethical guidelines were followed for the conduct of animal experiments in strict compliance with the Institutional Animal Ethical Committee (IAEC) and Committee for the Purpose of Control and Supervision of Experiments on Animals (CPCSEA), Government of India (IAEC-KV-8/2007–2508-BC-AA-9).

### Isolation of soluble and insoluble proteins

Decapsulated lenses were homogenized in ice-cold PBS (pH 7.4) in a glass homogenizer. The homogenate was centrifuged at 10,000× g for 20 min at 4 °C. The supernatant was collected, the pellet washed thrice with the same buffer and supernatant collected. The obtained supernatant comprised of the water-soluble lens proteins and designated as WSF. Obtained pellet was resuspended in 7 M Urea and 50 mM Tris-HCl (pH 7.4) diluted to 1:1. The homogenate was centrifuged at 10,000× g for 20 min, the supernatant collected and the procedure repeated thrice. The obtained supernatant comprised of the insoluble lens proteins and protein concentration was determined [21].

### Isolation and purification of lens crystallins

Lenses from 2 to 3 animals were pooled and homogenized in a buffer containing 0.025 M Tris, 0.1 M NaCl, 0.005 M EDTA, and 0.01% NaN_3_, pH 8.0 (TNEN buffer) and centrifuged at 10,000× g for 30 min at 4 °C to separate water soluble and water insoluble fractions. The water soluble fraction was applied onto a 90 cm×2.5 cm Sephacryl S-300 HR column for separating the crystallins and fractions were collected using fraction collector (Bio-Rad, Hercules, CA). Peaks corresponding to αH-, αL-, βH-, βL-, and γ-crystallins were pooled and dialysed extensively against the buffer. To avoid possible contamination of individual crystallins with other crystallins, 2–3 fractions between the peaks were discarded. Protein concentration was determined [21] and crosschecked by absorption at 280 nm. The purity of pooled crystallins was assessed by SDS–PAGE (Bio-Rad) and they were dialyzed against water and stored at −20 °C until further use.

### Chaperone activity of α-crystallin

The chaperone activity of α-crystallin was probed by measuring its ability to prevent the aggregation of substrate proteins denatured by reduction of disulfide bonds (insulin) or heat (βL-crystallin) [22]. Briefly, 0.4 mg/ml insulin in 50 mM phosphate buffer, pH 7.4 in the absence or presence of 0.5 mg/ml of α-crystallin was reduced with 20 mM DTT. Similarly, 0.3 mg/ml of βL-crystallin in 50 mM phosphate buffer in the absence or presence of 0.3 mg/ml α-crystallin was heated at 65 °C for 50 min. The aggregation of substrate proteins upon denaturation was measured by monitoring the light scattering at 360 nm as a function of time using spectrophotometer.

### Two-dimensional gel electrophoresis

Protein samples were mixed with loading buffer for the Isoelectric Pulse Gel (IPG) strips. The mixture was applied to dry 11cm immobilized pH 3–10 linear gradient strips (Ready Strip IPG strip; Bio-Rad). For SDS–PAGE, the IPG strips were incubated in equilibration buffer and the equilibrated IPG strips were transferred for the second dimension SDS–PAGE onto 12% gel. Electrophoresis was performed using a SE600 system (Amersham Pharmacia, Uppsala, Sweden). The Coomassie blue-stained gel images were captured and image analysis performed on computer (Melanie 3 software; Geneva Bioinformatics, Geneva, Switzerland) to determine the percent that each spot contributed to the total protein on the gel.

### Immunoblot analysis

Insoluble proteins of rat lens (40 mg/well) were separated on a 12% SDS-gel (Bio-Rad). The proteins were electroblotted onto a nitrocellulose membrane (0.45 mm; Bio-Rad) under cold condition for 1 h at 100 V. The membrane was blocked overnight at 4 °C with 2% BSA (BSA, Fraction V prepared in Tris buffered saline-Tween-20). After rinsing the membrane with Tris buffered saline Tween (TBST), it was incubated for 2 h with 1:1,000 diluted rabbit primary antibody (anti-vimentin and anti-tubulin). The membrane was washed three times (15 min each) with TBST and incubated with 1:5,000 diluted goat anti-rabbit IgG secondary antibody coupled to alkaline phosphatase for 1 h. The membrane was washed three times again (15 min each) with TBST buffer and developed with the BCIP/NBT substrate which developed into purple blue insoluble precipitates indicating the presence of vimentin and tubulin. β-Actin was used as a loading control with rabbit anti-β-actin primary antibody at 1:1,000 dilution.

### Scanning electron microscopic (SEM) studies

Dissected lenses were immediately fixed in 3% glutaraldehyde in 0.1 M phosphate buffer at 4 °C. After fixation the lenses were dehydrated in graded acetone series and critical point dried. The lenses were then coated with gold and used for the SEM study under a Philips scanning electron microscope 501(B) at 50 kV (Philips, Hillsboro, OR), and suitable photographs were taken. Samples were processed and sectioned by the method of Anderson and Shearer [23].

### Transmission electron microscopic (TEM) studies

Lenses were fixed in 2.5% glutaraldehyde and 2% paraformaldehyde for 12–18 h. Samples were processed and sectioned by the published method  [24] Briefly, lenses were fixed in 2.5% glutaraldehyde and 2% paraformaldehyde for 12-18 h. Samples were processed, sectioned, stained and examined under a Hitachi H-600 Transmission electron microscope (Hitachi, Krefeld, Germany)..

### Statistical analysis

All statistical calculations were performed with Statistical Package for Social Sciences (SPSS; IBM-SPSS Statistics, Armonk, NY) software program. The values are expressed as the mean±SD. The data were statistically analyzed using ANOVA (ANOVA) and significant difference of means was determined using Duncan’s multiple range tests at the level of p<0.05.

## Results

### Soluble and insoluble proteins

**Figure 1:**
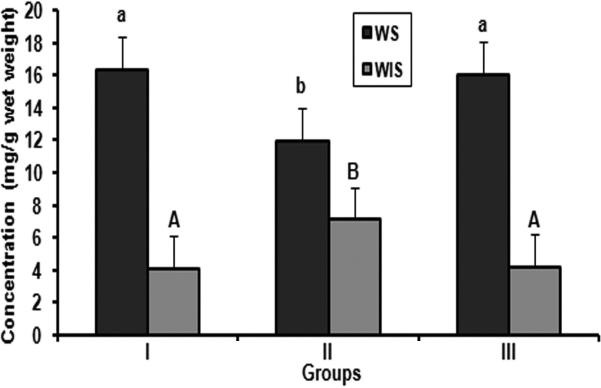
Soluble and insoluble proteins in lens. Values are expressed as mean (n=8) ±SD. Comparison between groups, different alphabets indicate significant difference at p<0.05. Groupings are G-I: Control, G-II: Selenite induced, G-III: Selenite + FVN treated.

Whatever is the underlying mechanism, alteration in protein profile and insolublization of soluble protein has been considered to be the ultimate factor in lens opacification. Therefore, we analyzed the soluble and insoluble protein content in all the three groups. In control rats, no significant changes were observed in the ratio of soluble to insoluble proteins (Figure 1). In contrast, the ratios of soluble to insoluble proteins were altered in selenite induced group. A significant decrease of soluble and increase of insoluble proteins were observed compared with control (Figure 1). No significant changes in the loss of soluble to Insoluble protein ratio were found in the FVN treated group compared with control (Figure 1).

### Gel filtration profile of crystallins

**Figure 2:**
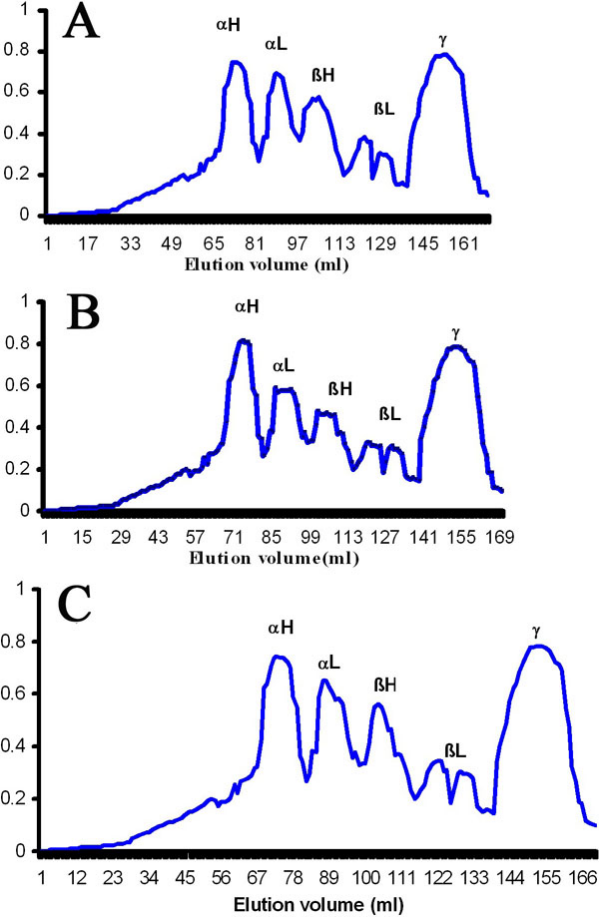
Gel filtration profiles of crystallins. Elution profiles of water soluble proteins from the experimental groups using Sephacryl - 300HR size exclusion chromatography. **A**: Control, **B**: Selenite induced, **C**: Selenite + FVN treated. αH, αL, βH, βL, and γ represents the corresponding crystallins elution peaks. Data are representative of three such independent assays for three separate lens extracts.

To investigate possible alterations in crystallins profile of selenite cataract and the influence of *V. negundo*, the soluble proteins were analyzed by Sephacryl - 300HR size exclusion chromatography. Data are representative of three such independent assays for three separate lens extracts. Analysis of crystallin profile suggests that the quality of the existing soluble proteins varied between Group II and Group I (Figure 2A,B). The elution profile of selenite induced group showed a slight increase of αH- and a decrease of αL-, βH-, and βL-crystallin peaks compared with control. However, there was no difference in the γ-crystallin peak in these groups (Figure 2A,B). Nevertheless, all these modifications were prevented by FVN treatment (Figure 2C).

### Chaperone activity of α-crystallin

**Figure 3:**
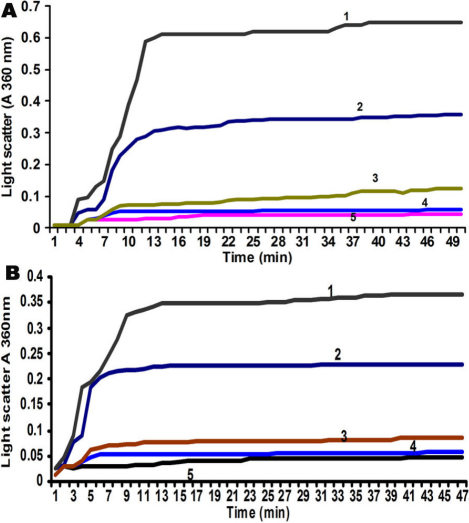
Chaperone activity of α-crystallin. **A**: DTT induced aggregation of insulin - Light scattering of insulin in the absence of α-crystallin (curve 1), α-crystallin from selenite induced (curve 2), α-crystallin from control rats (curve 4), standard α-crystallin (curve 5), α-crystallin from FVN treated group (curve 3). Data are representative of three such independent assays for three separate lens extracts. **B**: Heat induced aggregation of βL-crystallin - absence of α-crystallin (curve 1), α-crystallin from selenite induced group (curve 2), α-crystallin from control (curve 4), standard α-crystallin (curve 5), α-crystallin from FVN treated group (curve 3). Data are representative of three such independent assays for three separate lens extracts.

This was performed with α-crystallin isolated from control, selenite induced, and *V. negundo* treated rats by two methods. In DTT induced aggregation of insulin assay, reduction of disulfide bonds connecting insulin A- and B-chains leads to the unfolding and aggregation of the B-chain. This aggregation is suppressed by α-crystallin, which binds to a non-native conformer of the B-chain. Data are representative of three such independent assays for three separate lens extracts. Figure 3A shows the relative chaperone activity of α-crystallin isolated from the three groups. Light scattering of insulin was increased in the absence of α crystallin (Figure 3A, curve 1). Chaperone activity of α-crystallin isolated from selenite induced group was diminished (Figure 3A, curve 2), but this aggregation was completely suppressed by α crystallin isolated from control rats (Figure 3A, curve 4) compared with standard α crystallin (Figure 3A, curve 5). However, protection from aggregation increased markedly using α-crystallin from FVN treated group (Figure 3A, curve 3) compared with control. In heat induced aggregation of βL-crystallin assay, as shown in Figure 3B aggregation of βL-crystallin was increased at 60 °C in the absence of α crystallin (Figure 3B, curve 1). The chaperone activity of α-crystallin isolated from selenite induced group showed a marginally decreased activity (Figure 3B, curve 2) compared with control (Figure 3B, curve 4). However, improved chaperone function of α-crystallin was found in FVN treated group (Figure 3B, curve 3).

### Lenticular proteomics and 2D-PAGE

**Figure 4:**
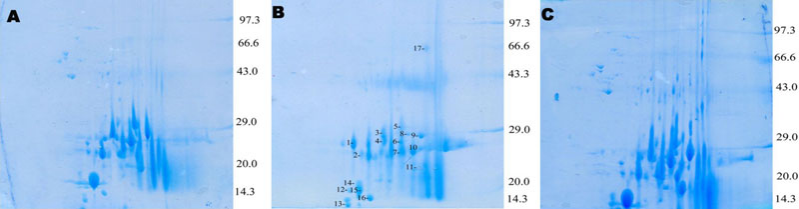
Lenticular proteomics and 2D-PAGE. **A**: Control, **B**: Selenite induced, **C**: selenite + FVN treated. 2D-PAGE map of selenite induced group showed few spots are newly visible, few spots intensified and another few diminished in intensity or had disappeared (marked in alphabetical order from 1 to 17). FVN treated group showed normal appearance in 2D-PAGE analysis compared with control.

To resolve the modification in lens proteins of selenite cataract and the influence of *V. negundo*, the soluble proteins were analyzed by 2D-PAGE. Selenite-induced cataract is a useful model to study the insolubilization of lens protein during cataractogenesis and 2D-PAGE is a valuable tool for this. In our previous study, we used SDS–PAGE to characterize the modification to lens protein. The data obtained from the 2D-PAGE map it is clear that few spots are newly visible, few spots intensified and another few diminished in intensity or had disappeared (Figure 4, marked in alphabetical order from 1 to 17) in the selenite induced group. *V. negundo* treated group showed normal appearance in 2D-PAGE analysis compared with control.

### Immunoblot analysis of cytoskeletal proteins

**Figure 5:**
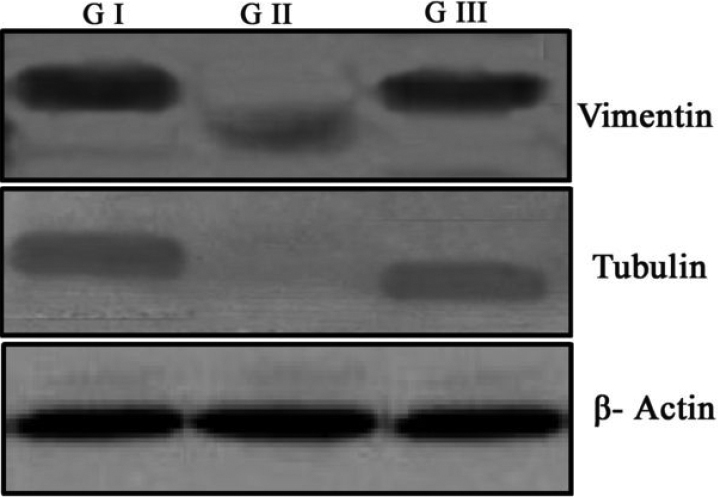
Immunoblot analysis of cytoskeletal proteins. Selenite induction (G II) accelerated the loss of cytoskeletal proteins tubulin and vimentin in selenite induced group. Treatment with FVN (G III) prevented the loss of cytoskeletal proteins compared to control (G I). β-Actin was used as a loading control.

Cytoskeletal proteins such as the vimentin-acin-tubulin system may stabilize the transparent cell structure. In our study (Figure 5), selenite induction accelerated the loss of cytoskeletal proteins tubulin and vimentin in selenite induced group. Treatment with FVN prevented the loss of cytoskeletal proteins in Group III compared to control (Figure 5).

### Scanning electron microscopic changes

**Figure 6:**
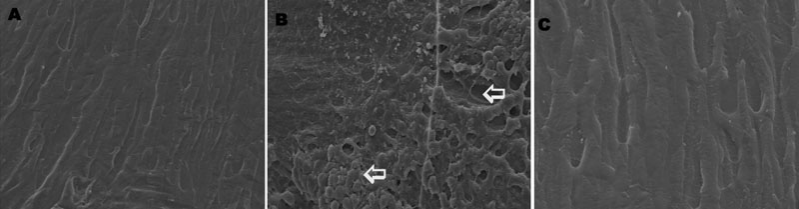
Scanning electron microscopic changes. **A**: Control, **B**: Selenite induced, **C**: Selenite + FVN treated. Dissected lenses were fixed in 3% glutaraldehyde in 0.1 M phosphate buffer at 4 °C. After fixation the lenses were dehydrated in graded acetone series and critical point dried. The lenses were then coated with gold and used for the SEM study under a Philips scanning electron microscope 501(B) at 50 kV. Magnification – 2,000×. Arrows indicates abnormal fiber structure and numerous spherical bodies in lens.

Scanning electron microscopy studies in selenite induced group showed abnormal fiber structure, apparent fusion of membranes and numerous spherical bodies in lens. Normal appearance was obtained in FVN treated group compared to control (Figure 6).

### Transmission electron microscopic changes

**Figure 7:**
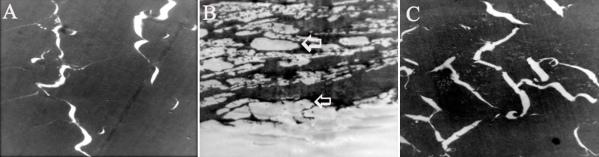
Transmission electron microscopic changes. **A**: Control, **B**: Selenite induced, **C**: Selenite + FVN treated. Lenses were fixed in 2.5% glutaraldehyde and 2% paraformaldehyde for 12–18 h. Samples were processed, sectioned, stained and examined under a Hitachi H-600 Transmission electron microscope. Magnification – 8,400×. Arrows indicates enlarged, irregularly shaped fibers with opaque material.

Transmission electron microscopic appearance showed strikingly difference between control and selenite induced groups. Nuclear region of the selenite induced lenses contained enlarged, irregularly shaped fibers, each of which had a lacy appearance. In the most central regions, only opaque material remained in each lens fiber. In FVN treated group electron microscopic examinations revealed reduction in the pathological changes in lens (Figure 7).

## Discussion

Oxidative stress has long been recognized as an important mediator in the pathogenesis of cataract [25] whereas lens exists in an environment rich in ROS. There is substantial evidence that oxidative stress is partly responsible for selenite cataract and antioxidant can be effective inhibitors of cataractogenesis in selenite model [10]. Several studies including ours suggest that medicinal plants having antioxidant potential could protect the system against the toxic effects of ROS [11-17]. Our laboratory as well as others has reported the free radical scavenging potential *V. negundo* [13,26,27]. Generation of ROS implicated with degradation, cross linking and aggregation of lens proteins [28].

In our previous study we have reported the mechanism of action of the flavonoids from *V. negundo* in selenite cataract [13]. *V. negundo* exerts its effect by preventing oxidative stress and calpain activation thereby protecting over selenite cataract. This work is an extension of our previously published work and here we have studied various changes in lens proteins and structure during flavonoid treatment.

One of the major events in the selenite cataract is the calpain mediated proteolysis of lens crystallins. This partially degraded α- and β-crystallins becomes insoluble and scatter light [10]. Loss of soluble protein in selenite induced group in our study could be due to proteolysis and insolublization, which may be important during the early stages of opacfication. A fivefold increase in the amount of insoluble protein in selenite cataract has been reported previously [29,30]. The ability of *V. negundo* to prevent the loss of soluble protein is remarkable (Figure 1). *V. negundo* not only prevented the decrease in soluble proteins but also prevented crosslinking/aggregation and insolublization. This may be due to protection over oxidative stress and calpain mediated proteolysis [13], which is in agreement with the delay of cataract maturation in this group. Our result agrees well with the reported data [15,31].

Maintenance of crystallin profile is essential for lens transparency and the alteration in elution profile in selenite induced group may be due to the proteolytic degradation of crystallins. This is consistent with the earlier reports [32] which indicated that proteolysis had resulted in an increase in αH-crystallin and decrease of αL-, βH-, and βL-crystallins and loss of many polypeptides from the soluble, insoluble, and intrinsic membrane fraction. Treatment with FVN prevented the loss of crystallin profile, which may be due to less calpain activation in this group (Figure 2C). Similar results were reported in experimental cataract [33].

It has been proposed that conformational changes and unfolding of proteins in the lens are due to post translational modifications [6], which may be responsible for the decrease of α-crystallin chaperone activity in selenite cataract [34]. Chaperone activity has been proposed to be a major function of α-crystallin, which was studied by assessing its ability to prevent the aggregation of substrate protein denatured by either reducing agent or heat. Therefore, it is of considerable importance to investigate the protective effect of antioxidants on α-crystallin chaperone activity. Chaperone activity of α-crystallin isolated from selenite induced group was diminished and protection from aggregation increased markedly using α-crystallin from FVN treated group, which indicates its protection over the lens crystallins (Figure 3A). In heat induced aggregation of βL-crystallin assay, chaperone activity of α-crystallin isolated from selenite induced group showed a marginally decreased activity and better chaperone function of α-crystallin from the FVN treated group, which further confirms its protective role. Thus, one of the possible explanations for the modulatory effect of FVN on α-crystallin chaperone activity in selenite cataract (Figure 3B) could be due to decreased oxidative stress and calpain activation. Earlier reports on disrupted α-crystallin chaperone activity by calpain activation lend support to our explanation [34,35].

2D-PAGE is an emerging tool for proteomics which holds great promise in cataract research [6]. These 2D-PAGE gels should be useful to examine rapidly other post-translational modifications and decrease chaperone activity of α-crystallins, possibly leading to the observed increases in insoluble proteins during selenite cataract [35]. Loss of chaperone activity of α-crystallin during selenite cataract has been studied using this technique [34]. This technique also help to determine the proteolysis of crystallins associated with insolubilization of proteins in rat lens during maturation of selenite cataract and implication of calpain II [36,37]. So it has much significance in cataract research and has been included in this study. In our study, from 2D-PAGE map it is clear that few spots are newly visible, few spots intensified and another few diminished in intensity or had disappeared in selenite induced group. During aging and cataract formation, insolubilization of soluble proteins in the human lens is increased [10]. The primary cause of insolubilization is unknown, but it may be due to post-translational modifications of lens crystallins. Selenite-induced cataract is a useful model to study the insolubilization of lens protein and 2D-PAGE is a valuable tool for this [29].

The possible reasons for the post translational modification in selenite cataract are oxidative stress and calpain mediated proteolysis [38]. Oxidative damage due to selenite induction inactivates membrane Ca^2+^ATPase leads to Ca^2+^ accumulation and subsequent activation of calpains, which partially degrade the crystallins and there by resulting in the protein insolubilization [35]. This postulated mechanism confirms the results of our previous study [13]. Alterations in selenite induced group are due to insolubilization and formation of urea soluble proteins in lens. *V. negundo* treated group showed normal appearance in 2D-PAGE analysis compared to control which may be due to its protective effect (Figure 4). The results are inconsistent with the earlier reports [39].

Cytoskeletal proteins in the lens account for only a small proportion of total protein, but they are thought to play a significant role in the maintenance of lens transparency, particularly in the stabilization of the fiber cell during and after differentiation when the fiber cell undergoes massive changes in its dimensions [40]. Additionally, cytoskeletal proteins may play an important role in the facilitation of the chaperone function of α-crystallin in the lens, which is thought to be crucial in maintaining optical clarity in the lens [41]. Calpain mediated proteolysis of the cytoskeletal proteins may disrupt the interaction between crystallins and the cytoskeletal proteins [10]. This is because cytoskeletal proteins are especially good substrates for calpain and seems to be the first protein lost in selenite cataract [31]. Thus, degradation of cytoskeletal proteins may be an important mechanism during early stage of selenite cataract. Previous studies showed that 24 h after the injection of selenite, the ratio of insoluble to soluble protein increased as lens opacification began [29]. It may also be noted that the inappropriate loss of cytoskeletal proteins within 72 h post induction corresponds to the abnormal variation in phase separation temperature, membrane damage and loss of fiber structure [42]. These reports indicate that properly organized cytoskeleton may be necessary for normal development and maintenance of lens transparency. Treatment with FVN prevented the loss of cytoskeletal proteins, which further confirms its protective effect (Figure 5). Our results are in consistent with the earlier reports [31].

SEM study was performed to localize morphological changes in the lens of experimental groups, which indicates the damage probably due to the loss of proteins [23]. This showed the loss of cytosol within affected fibers where the remaining contents were clumped and irregular. This contrasted sharply with fibers of control and treated lenses in which the membranes remained intact. Selenite cataract is produced within a few days of injection and the age of the fibers is a major factor in its susceptibility to selenite stress. The newly formed fibers push the deformed superficial fiber formed during selenite stress into the adult nuclear area where they form opacity. This process is accelerated by the calpain system [37]. Eventually it results in loss of fiber boundaries. Spherical bodies similar to those found in the nuclear region have been reported in several human and experimental cataract [43-45]. Calpain mediated proteolysis is important in selenite cataractogenesis and may be part of the mechanism which produces spherical bodies in lens [29]. In the treated groups, reduction in the pathological changes observed may be due to the protective effects of FVN (Figure 6).

One of the most outstanding features of selenite cataract is the rapid and significant increase in the amount of insoluble protein in the lens [46]. Electron microscopic studies of selenite model revealed opaque aggregates in the cell matrix, which may be due to precipitation of cytoplasmic proteins. Previous transmission electron microscopy pictures of selenite nuclear cataract showed formation of a two-phase system and aggregation of the cytoplasmic protein onto the membranes [47]. Thus, it is likely that the lacy networks observed in the nuclear fibers and the cytoplasmic aggregations seen just inside the membrane are insoluble protein. A similar increase in insoluble material accompanied by an increase in the granularity of the cytoplasm has been reported in experimental cataracts [48]. In the treated groups, reduction in the pathological changes observed may be due to the protective effects of FVN (Figure 7).

In the present study, intraperitoneal administration of FVN prevented the alterations in lens proteins during the course of selenite cataract, further supporting our previous reports. We have employed various proteomic tools to elucidate the post translational modifications in selenite cataract with FVN and this seems to be the first report on this aspect. In selenite induced group level of soluble proteins and chaperone property of α-crystallins were altered. In 2D-PAGE analysis we have identified various modifications and loss of cytoskeletal proteins as demonstrated by immunoblot analysis. On the other hand treatment with FVN effectively modulated these changes in lens proteins, exhibiting better anticataractogenic potential.
